# Traditional Chinese herbal medicine: harnessing dendritic cells for anti-tumor benefits

**DOI:** 10.3389/fimmu.2024.1408474

**Published:** 2024-09-19

**Authors:** Mengyi Shen, Zhen Li, Jing Wang, Hongjie Xiang, Qi Xie

**Affiliations:** ^1^ Department of Oncology, The First Affiliated Hospital of Shandong First Medical University & Shandong Provincial Qianfoshan Hospital, Shandong Lung Cancer Institute, Jinan, China; ^2^ Shandong First Medical University & Shandong Academy of Medical Sciences, Jinan, China; ^3^ Department of Traditional Chinese Medicine, The First Affiliated Hospital of Shandong First Medical University & Shandong Provincial Qianfoshan Hospital, Jinan, China; ^4^ School of Preventive Medicine Sciences, Shandong First Medical University & Shandong Academy of Medical Sciences, Jinan, China; ^5^ College of Traditional Chinese Medicine, Shandong Second Medical University, Weifang, China

**Keywords:** Chinese herbal medicine, dendritic cells, maturation, anti-tumor, immunity

## Abstract

Chinese Herbal Medicine (CHM) is being more and more used in cancer treatment because of its ability to regulate the immune system. Chinese Herbal Medicine has several advantages over other treatment options, including being multi-component, multi-target, and having fewer side effects. Dendritic cells (DCs) are specialized antigen presenting cells that play a vital part in connecting the innate and adaptive immune systems. They are also important in immunotherapy. Recent evidence suggests that Chinese Herbal Medicine and its components can positively impact the immune response by targeting key functions of dendritic cells. In this review, we have summarized the influences of Chinese Herbal Medicine on the immunobiological feature of dendritic cells, emphasized an anti-tumor effect of CHM-treated DCs, and also pointed out deficiencies in the regulation of DC function by Chinese Herbal Medicine and outlined future research directions.

## 1 Introduction

Cancer has always been a problem with high morbidity and mortality rates. Patients with cancers require multi-disciplinary integrated diagnosis and therapy (1, 2). This encompasses the combination and planning of diverse treatment modalities (3–7). Cancer treatments lead to various side effects. These can range from immune-related adverse events impacting the skin, and the system of gastrointestinal and endocrine to manifestations such as nervous and hematopoietic toxicities (8), furthermore, these treatments induce adverse reactions on organs such as the liver, pancreas, and heart (9, 10). As the result, cancer patients often experience a shorter survival time and a lower quality of life.

Cancer immunotherapy has emerged as a standard therapeutic approach, and has been combined with many new technologies to improve efficacy. For example, DNA sequencing detects tumor-specific antigens to develop tumor vaccines (11). Or based on radiomics to better evaluate tumor clinical outcomes and immunosuppressive response (12). Tumor immunotherapy is categorized as active or passive. One of active immunotherapy takes advantage of antigen-presenting cells (APCs), for instance dendritic cells (DCs) to enhance the patient’s immune response to kill tumors (13–15). DCs act as the primary APCs *in vivo* and play a vital part in activating anti-tumor immune response.

The effectiveness of cancer therapy is often hindered by multidrug resistance exhibited by cancer cells (16, 17), thus researchers and clinicians are constantly searching for more effective treatments. Chinese Herbal Medicine (CHM) has long been utilized to heal patients with cancers in China. It not only improves clinical symptoms but also enhances quality of life for cancer patients with minimal side effects (18). CHMs exert anti-tumor effects by upregulating immune responses in tumor microenvironment (19). Data have shown that CHM obviously boosted sensitivity to chemotherapy drugs and considerably ameliorate tumor-related fatigue, myelosuppression, and else adverse reactions (20). A growing body of evidences suggest that CHM and its active ingredient can affect immune responses at an early stage by targeting functions of DCs. In summary, the current cutting-edge research on CHM for cancer treatment includes phenomics, the anti-tumor effects of CHM monomers or compound formulas, and the combined application of CHM with modern therapies (21). However, the depth and breadth of mechanism studies are insufficient. For instance, systematic reviews are needed to investigate the regulatory effects of CHM on the immune system.

## 2 Subsets of dendritic cells and their functions

Dendritic cells (DCs) play an important role in innate immune system, by processing endocytosed pathogenic microorganisms. Through combining with MHC-I or MHC-II molecules, the antigenic peptides are further presented to the B and T cells, initiating the immune response of B and T cells (22). Immature DCs (iDCs) found in boundary possess a strong phagocytic capacity to capture apoptotic and necrotic cells (23). iDCs turn into mature DCs (mDCs) upon exposure to various activation through combining with pattern recognition receptors (PRRs) which are characterized by enhancing surface expression of MHC molecules and co-stimulatory molecules (CD86, CD80 and CD40). Additionally, mDCs secrete multiple pro-inflammatory cytokines, enabling them to effectively activate effector lymphocytes (24, 25).

DCs are categorized into different subgroups. Those are originated from common DC forerunners in the bone marrow, and are generally classified into conventional DCs (cDCs) and plasmacytoid DCs (pDCs). The precursor cDCs can further differentiate into type II cDCs (cDC2) and type I cDCs (cDC1) (26). Inflammatory situations can give rise to the CC-chemokine receptor 2 (CCR2)-dependent recruitment of monocytes from the blood, which then divide into monocyte-derived DCs (MoDCs) in peripheral tissues (27, 28).

### 2.1 cDC1

cDC1s, particularly in humans, are characterized by the presence of CD141 on their cell surface. Migratory subsets exist in lymph nodes and peripheral tissues (29). The cellular indication markers that make out cDC1 are Clec9A^+^, Sirp (CD172)^−^, CD11c^+^, CD11b^−^, CD141^+^, CD123^−^ and HLADR^+^ (26). cDC1 is chiefly responsible for antigen cross-presentation and plays an essential part in anti-viral and tumor immune responses. CD141^+^DC expresses Toll-like receptor 3 (TLR3) at high levels, produces INF-β and IL-12p70, induces superior Th1 response, and can sense pathogen-associated molecular patterns (PAMP) through TLR3. Moreover, poly I:C-actived CD141^+^DC has a high ability to cross-submit soluble protein antigen (Ag) to CD8^+^ cytotoxic T lymphocytes by MHC I, leading to inducing an adaptive immune response (30). IL-12 secreted by cDC1 can active NK cells to produce IFN-γ (31). The cDC1 of mouse lymphoid organs chiefly expresses CD8α, Clec9a, Xcr1 on the surface. And in the non-lymphoid tissues, cDC1 expresses CD103 (32).

In recent years, drug targeting cDC1s as an anti-tumor therapy has achieved promising clinical efficacy. In animal and clinical trials, it has been found that B-cell lymphoma 2 (BCL2) can act as a specific immune checkpoint for cDC1s, activating cDC1s and thus promoting anti-tumor immunity (33, 34).

### 2.2 cDC2

The cell surface factors that make out cDC2 are Clec9A^−^, CD123^−^, HLADR^+^, CD11c^hi^ Sirp^+^ and CD1c^+^ (35–37). Myeloid cDC2 is similar to monocytes, and expressed extensive lectins, RIG-I-like receptors, Nod-like receptors and Toll-like receptors (27). They play a vital role in autoimmune diseases and anti-bacterial defense and in keeping the immune tolerance (38). cDC2 has been shown to induce Th1, Th2 and Th17 cells (39, 40). Two new subtypes of CD1c^+^ have been identified. Human CD1c^+^CLEC10A^+^CLEC4A^lo^ cDC2s and CD1c^lo^CLEC10A^–^CLEC4A^hi^ cDC2s, are the equal to mouse T-bet^-^cDC2s (‘cDC2B’) and T-bet^+^cDC2s (designated ‘cDC2A’), severally. T-bet^–^ cDC2s more likely to cause inflammation, while T-bet^+^cDC2s show transcripts encoding molecules participated in repairing of tissue and own a reduced capacity to polarize naive T cells (41). In mouse, cDC2 have a more heterogeneous expression of Sirpa, CD11b, and other on the surface (42, 43). James et al. found that in pancreatic adenocarcinomas (PDACs) patients, cDC2s were systematically suppressed by significantly increased IL-6, as IL-6 disrupted the polarization of cDC1s and cDC2s, impaired antigen processing and presentation functions, resulting in anti-tumor immunity being affected (44). Luo et al. found that blocking Tim-3 in cDC2 promoted CD4^+^T cell and enhanced the anti-tumor ability of ADU-S100(S100), an agonist for Stimulator of interferon genes (STING) (45).

### 2.3 pDC

pDC, which are found in blood and lymphoid tissues, and certain organs like the lung in mice and tonsils in humans (29). pDCs can be characterized by cell surface markers CD11c^lo^, CD123^+^, HLADR^+^, BDCA2 (CD303)^+^, BDCA4 (CD304)^+^, CD45RA^+^ (46, 47). After recognizing the virus or its own nucleic acid by Toll-like receptor 9 (TLR9) and TLR7 (47), pDC produces a significant amount of type I interferon (IFN) as well as a small amount of III IFN (48), acting as an antiviral agent (49, 50). Type I IFN is crucial for the cross-presentation of CD8α^+^ DC and the development of tumor antigen specific CD8^+^ T cell *in vivo* (51). pDC also produces various chemokines and cytokines like CC chemokine receptor 4 (CCL4), CCL3, CXC chemokine ligand 10 (CXCL10), CXCL8, IL-6 and IL-12. pDCs submit antigens to CD4^+^T cells through co-stimulatory molecules and MHC II (47). Cha et al. found the enrichment of pDCs was closely related to the prognosis of smoking-induced lung cancer (52).

### 2.4 MoDC

MoDCs, which reside in the skin, lung, and intestine, can be identified by specific cell surface markers including CD11c^+^, HLADR^+^, CD14^+^, CD11b^+^, CD1c^+^, CD209^+^, CD206^+^, CD64^+^, CCR2^+^, CD1a^+^ and CD172a^+^ (29). MoDC is primarily produced in response to inflammation and promotes environmentally dependent division of CD4^+^ T cells into the type 2 helper T cells (Th2 cells), type 1 helper T cells (Th1 cells), or IL-17-producing helper T cells (53). Additionally, MoDCs secrete numerous inflammatory cytokines and participate in the partial inflammatory response (54). Raccosta et al. found a new antagonist of the oxysterol receptors called Liver X Receptors (LXRs) promoted the differentiation of MoDCs within tumors and enhanced their anti-tumor effects in mice (55).

Here we summarize the subsets of dendritic cells in **Table 1**.

**Table 1:** Subsets of dendritic cells.

Name	Presence site	Surface markers	Main function
**cDC1**	peripheral tissues and lymph nodes	HLADR^+^, CD11c^+^, CD123^−^, CD11b^−^, Sirp (CD172)^−^, CD141^+^, Clec9A^+^	cDC1 is chiefly responsible for antigen cross-presentation and plays a crucial part in anti-viral and tumor immune responses.
**cDC2**	lymphoid tissues, blood, peripheral tissues and lymph nodes	HLADR^+^, CD11c^hi^, CD123^−^, Sirp^+^, CD1c^+^, Clec9A^−^	They play a vital part in anti-bacterial defense and autoimmune illnesses and in keeping the immunologic tolerance. cDC2 has been shown to induce Th1, Th2 and Th17 cells.
**pDC**	lymphoid tissues, blood, and certain organs like the lung in mice and tonsils in humans	CD11c^lo^, CD123^+^, HLADR^+^, BDCA2 (CD303)^+^, BDCA4 (CD304)^+^, CD45RA^+^	pDC produces a significant amount of type I interferon (IFN) and also a small amount of III IFN, acting as an antiviral agent.
**MoDC**	skin, lung, and intestine	CD11c^+^, HLADR^+^, CD1c^+^, CD11b^+^, CD14^+^, CD64^+^, CD206^+^, CD209^+^, CD172a^+^, CD1a^+^ and CCR2^+^	MoDC is primarily produced in response to inflammation and promotes environmentally dependent differentiation of CD4^+^T cells into type 2 helper T cells (Th2 cells), type 1 helper T cells (Th1 cells), or IL-17-producing helper T cells.

## 3 Alternation of antigen-presenting related molecular and cytokines in DCs treated with CHM

With advancements in technology, the active ingredients of CHM were ascertained and purified, which covered polysaccharides, saponins and more. Polysaccharides are the main active ingredient of CHM and have a series of biological activities, including anti-diabetes, anti-oxidant, anti-viral, anti-inflammatory, anti-tumor, hepatoprotection, immunomodulation, radioprotection and neuroprotection (56, 57). CHM and their constituents have the capacity to facilitate the maturation of immature DC (iDCs) by upregulating the expression of major histocompatibility complex I and II (MHC I/II) and co-stimulatory molecules. This process leads to a significant increase in the generation of pro-inflammatory cytokines, such as IL-6, TNF-α, IL-1β and IL-12. These effects can enhance the immune response against tumors (25).

### 3.1 Polysaccharides

The studies by G. Cai and colleagues demonstrated that Alhagi honey polysaccharide (AH) conferred various benefits on mice by promoting the maturation of dendritic cells (DCs) and enhancing the production of CCL20, which aggregates DCs. This aggregation induced the activation of B cells, CD8^+^ and CD4^+^ T cells. Additionally, AH augmented the secretion of secretory IgA (sIgA) to protect the intestine. This is accomplished either by stimulating Th cells to divide and produce cytokines or by directly activating DCs to release cytokines, thereby increasing the number of IgA^+^ cells, J chain, and pIgR in the gut. AH also significantly elevated the levels of short-chain fatty acids (SCFAs) in the caecum, thereby helping to regulate cyclophosphamide-induced intestinal immune dysfunction. *In vitro* experiments further revealed that AH markedly enhanced pIgR protein expression in Caco-2 cells and promoted the maturation of DCs to regulate immune responses (58). Comparing the immune effects of Echinacea purpurea polysaccharide (EPP) and sulfated EPP (sEPP) on chicken bone marrow-derived DCs (chBM-DCs), L. Yao et al. found that both EPP and sEPP enhanced the expressions of CD11c and CD80, improved the capability of chBM-DCs to promote the proliferation of allogeneic mixed lymphocytes, and significantly increased the levels of IL-2 and IFN-γ, while down-regulating the levels of IL-4 and IL-10 (59). Y. Wu et al. found that Glycyrrhiza polysaccharide extract 1 (GPS-1) enhanced the generation of IFN-γ and IL-4, and increased the ratio of CD3^+^CD4^+^ T and CD3^+^CD8^+^ T lymphocytes in the mice spleen. Further experiments revealed that GPS-1 promoted maturation and phagocytosis of DCs. Similarly, H. Zhou et al. (60) also demonstrated that GPS-1 was able to enhance the secretion of IL-12, TNF-α and IFN-γ as well as promoted the secretion of NO, IL-2, IL-1β, IFN-β, TNF-α and IL-12p70 from DCs (61). In studies conducted by Y. Wu et al., the experiments *in vitro* showed that the acidic Epimedium polysaccharide (EPS-1) increased the proliferation of the splenic lymphocytes and the production of cytokines (IFN-γ, IL-2, TNF-α and IL-4). Furthermore, the expression of surface molecules of mature chBM-DCs (MHC II, CD11c, CD40 and CD86) and the levels of cytokines (IL-10 and TNF-α) were increased, and EPS-1 also enhanced phagocytosis rate of mature chBM-DCs (62, 63). Y. Zou et al. have observed that purified Achyranthes bidentata polysaccharide (ABP) promoted the upregulation of MHC II, CD40 and CD86 expressions, inducing the maturation of DC phenotypes. Moreover, ABP was demonstrated to enhance the production of IL-12 (64). X. Wang et al. found that Isatis root polysaccharide (IRPS) promoted the maturation of MoDCs, induced the secretion of IL-12, and reduced the expression of IL-6 (65). Y. Huang et al. found that Rehmannia glutinosa polysaccharide (RGP) significantly stimulated lymphocyte proliferation, and enhanced the production of IFN-γ and IL-2. The antigen presenting ability of DCs was also improved by RGP stimulation (66). J. Gao et al. found that Plantain polysaccharide (PLP) exhibited the ability to promote the maturation of DCs both *in vivo* and *in vitro*. In mouse model of breast tumors 4T1, PLP effectively controlled tumor growth and enhanced immune response by recruiting DC, CD8^+^T and CD4^+^T cells to the tumor microenvironment (67). M. Tian et al. found that Huangqi Guizhi Wuwu Tang (HGWT) displayed potent anti-tumor effect. Polysaccharides derived from Astragalus membranaceus and Polyporus umbellatus were demonstrated to promote DCs maturation (68).

### 3.2 Saponins

X. Zhao et al. discovered that Salidroside liposome effectively promoted the maturation of DCs. Additionally, it was found to stimulate MLR proliferation and enhance antigen presentation. Furthermore, Salidroside liposome was able to induce sustained cellular and humoral immune responses (69). C. Mo et al. found that Ginsenoside-Rg1 (G-Rg1) exerted its anti-fibrotic properties by reducing DC maturation mediated by IDO1 (70). H. Guo et al. found that Astragaloside IV (ASI) effectively promoted the maturation of DC phenotype, by means of significant upregulation of CD14, CD40, CD80, CD83, CD86 and HLA-DR. Additionally, ASI treatment enhanced the release of IL-12, thereby bolstering the immune response (71).

### 3.3 Others

S. Nabeshima et al. found Hochu-ekki-to (HOT) stimulated DCs to mature in a dose-dependent manner by increasing the expression of CD83, CD86, CD80 and by producing IL- 12 (72). Y. Fu et al. found that Myrothecine A was able to promote the expression of CD86 and CD40 on DCs (73). D. F. Huang et al. discovered that the Plantago asiatica L. seeds extract (ES-PL) induced DC maturation with high level of MHC II and key stimulatory molecules CD80 and CD86. Functional maturation of ES-PL-treated DC was demonstrated by reduced mannose receptor-mediated endocytosis and strengthened antigen presentation to allogeneic naive or homogeneously activated T lymphocytes. Additionally, the gene expression of *CCR7* in ES-PL-treated DC was also enhanced (74). C. Y. Li et al. observed that Cordyceps sinensis had the ability to promote DC maturation. Cordyceps sinensis was found to stimulate the expression of costimulatory molecules on DCs, to increase the expression of the inflammatory factors, and to enhance the proliferation of allogeneic T cells. These findings suggested that Cordyceps sinensis has the potential to strengthen DC function and stimulate anti-tumor immune responses (75). N. Takeno et al. discovered that Shi-Quan-Da-Bu-Tang (SQDBT) enhanced OVA antigen phagocytosis and antigen presentation in DCs *in vitro*. In addition, in mice inoculated with mouse lymphomas expression tumor antigens, the researchers observed a significant decrease in tumor growth and a prolongation of survival when SQDBT was administered (76). M. Kaneko et al. found that Hochu-ekki-to (Bu-Zhong-Yi-Qi-Tang) (HOT) significantly reduced OVA-specific IgG1 and IgE serum levels and antigen-specific proliferation of the cells in splenic organ of young mouse. HOT increased the amount of CD4^+^T cells and enhanced expressions of MHC II and CD86, CD80 and CD40 on antigen-presenting cells (77).

The researchers mentioned above have largely explored the anti-tumor properties of CHM, emphasizing the augmentation of MHC I and II expression, CD40, CD80, and the secretion of specific inflammatory factors. However, several important considerations arise. Firstly, there has not been a more detailed examination of the signaling pathways implicated in these processes. Secondly, the majority of these studies are conducted *in vitro* and focus on the modulation of DC function by CHM. Only a limited number of studies have been extended to animal models, and there is a significant absence of clinical trials to confirm these observations. These issues certainly merit our thorough attention and further investigation.

## 4 Pathway of CHM in regulating DC function

With further research, it has been discovered CHM primarily promotes the maturation of DCs through Toll-like receptors (TLRs), nuclear factor kappa-B (NF-κB) pathways, and Mitogen-Activated Protein Kinase pathway (MAPK). This activation leads to the secretion of pro-inflammatory cytokines, contributing to a more effective anti-tumor effect.

### 4.1 TLR pathway

TLRs play a crucial part in the activating innate immunity by recognizing specific microbial signatures, and are essential components of both the innate and adaptive immune systems. A series of studies have shown that TLRs signaling pathways were composed of two main components. The first is the MyD88-dependent pathway shared by all TLRs. The second is the MyD88-independent pathway specific to the TLR3- and TLR4 pathways (78). TLRs are widely expressed in various immune cells, and apart from tumor-infiltrating immune cells, other tumor also exhibit TLR activation (79, 80). Within the TLR family, TLR4 is particularly important for the maturation of DCs. MyD88 (myeloid differentiation primary response gene 88), which contains TIR (Toll-interleukin-1 receptor) domain, is an adaptor protein involved in the TLR4 pathway. TRAF6 (TNF receptor-associated factor 6), another adaptor protein, is crucial for the recruiting of IRAK1 in MyD88-dependent pathway (81). MyD88 activates TRAF6 and the downstream transcription factor NF-κB, thus promoting the generation of inflammatory factors.

Pearce et al. found that the activation of TLRs led to an early rapid increase in the rate of glycolysis within DCs. This metabolic shift supported the expansion of the Golgi and endoplasmic reticulum apparatus in DCs, which was necessary to accommodate the enhanced transport, protein synthesis, and cytokine secretion that were crucial for DC activation (82). J. J. Kim et al. found that the B-chain of Korean mistletoe lectin (KML-B) increased the expression of co-stimulatory molecules (MHC II, CD86, CD80 and CD40) and the secretion of cytokines (TNF-α, IL-12p70, IL-6 and IL-1β). Furthermore, KML-B influenced other markers of BMDCs maturation such as antigen uptake and CCR7 expression. The capacity of KML-B to improve BMDC features was depend on the expression of TLR4. Besides, naive CD4^+^T cells were directly or indirectly induced to differentiate into Th1 cells by KML-B matured BMDCs (83). S. Tanaka et al. proved that Chrysanthemum coronarium L. (C. coronarium) edible plant extracts significantly activated immune response through TLR9-, TLR4- and TLR2-dependent way. Stimulation with C. coronarium extract immediately activated CD11c^+^ DCs, leading to DC maturation characterized by increasing the expression levels of MHC II, MHC I, CD86, and CD40, and the production of IL-12 (84). X. Duan et al. found that Lycium barbarum Polysaccharides (LBP) promoted mature DCs in mice through TLR4-Erk1/2-Blimp1 signaling pathway. They discovered that LBP treatment induced DC maturation by upregulating MHC II and co-stimulatory molecules (CD80, CD86) and increasing the generation of IL-6 and IL-4 (85). X. Li et al. found that Radix Glycyrrhizae polysaccharide (GP) strengthened the generation of MHC I-A/I-E, CD86 and CD80 on DCs. It also facilitated the proliferation of allogenic CD3^+^T cells and the secretion of IFN-γ, while inhibiting the endocytosis of DCs on FITC-dextran. Additionally, GP increased the production of IL-12p70 in DCs in a time-dependent manner. Of note, the increase in IL-12p70 production was strongly suppressed by NF-κB, TLR4, p38 MAPK or JNK inhibitors, suggesting that GP was partially recognized by TLR4, and activation of MAPKs and NF-κB pathways may led to enhancing DC maturation (86). J. Du et al. investigated the effect of Actinidia eriantha Benth polysaccharide (AEPS) on DCs. They observed that AEPS decreased the BMDC phagocytosis and enhanced the expression of co-stimulatory molecules. AEPS also increased cytokines (IL-12p40, IL-10, IL-6, TNF-α, IL-1β, IFN-β and IFN-γ) and chemokines (CCL5, MIP-1α, MIP-1β, MDC and MCP-1). AEPS primarily exerted its effects through the TLR2/4 and NF-κB pathway to enhance DC maturation and functionality, thereby augmenting anti-tumor immune responses. The study also revealed elevated expression levels of STAT1, STAT2, and STAT5b, indicating the influence of JAK-STAT signaling on DC function (87). R. Bo et al. found that through TLR4 signaling pathway, Lycium barbarum polysaccharides liposomes (LBPL) significantly enhanced the proliferation of mouse DC precursor cells, and upregulated the expression of co-stimulatory molecules (CD86, MHCII and CD80) and cytokines (TNF-α, IL-12p40) to promote DCs maturation. The study further confirmed that LBPL upregulated the expression of *TRAF6, MyD88, TLR4, NF-κB* genes, and proteins (88). J. K. Wang et al. found that Matrine strengthened the secretion of inflammatory cytokines mediated by the TLRs signaling pathway to exert an anti-tumor effect. Matrine drastically enhanced the mRNA expression of *TLR8, TLR7, MyD88, IκB kinase* (*IKK*) and *TRAF-6*, as well as the protein expression of TLR8 and TLR7. In addition, Matrine also strengthened the expression of MHC-II, CD40, CD86, CD80 and CD54 in DCs, and stimulated the secretion of IL-6, IL-12 and TNF-α (89). Y. Tian et al. found that Astragalus mongholicus (AMs) promoted the expression of CD11c and DCs maturation in a dose-dependent manner. This effect was mediated by up-regulating TLR4 and inhibiting IκB-α (90). D. Wang et al. have summarized the impacts of Astragalus polysaccharide (APS) on immune cells. They found that APS significantly increased the proliferation of CD8^+^ T and CD4^+^ T cells and enhanced the expression of CD40, CD80, and CD86 in DCs. APS also enhanced the production of IL-12 and TNF-α, and induced the Th1/Th2 shift towards Th1 (32) (91). Zhou et al. found that APS activated DCs through the MyD88-dependent signaling pathway mediated by TLR4. When the TLR4 was activated by ligand, the TIR domain of the MyD88 protein was bound to the TIR domain of TLR, activating downstream TRAF-6. This, in turn, activated NF-κB, facilitating its entry into the nucleus and the subsequent activation of transcription of genes related to DC maturation. The activated DCs then expressed co-stimulatory molecules and related cytokines (92). J. Li et al. found that after treatment with Pleurotus ferulae water extract (PFWE), BM-DCs dose-dependently regulated the expression of CD86, CD80, CD40, and MHC II, and also strengthened the production of TNF-α, IL-6, and IL-12, primarily mediated by the TLR4 signaling pathway. Moreover, PFWE induced DCs to enhance the proliferation of allogeneic CD8^+^ T cells and improved their capacity to present antigens to autologous CD8^+^ T cells (93). T.-Y. Jung et al. found that Uncaria rhynchophylla ursolic acid extract enhanced the generation of CD83, CD80, CD1a, CCR7, HLA-DR and CD86 on DCs, promoted the proliferation of T cells, and strengthened the secretion of IFN-γ and IL-4. The production of IL-12p70 was induced by TLR2 and TLR4 signaling pathways, thereby inducing T cell differentiation to Th1 subtype (94). In addition, K. S. Kim et al. also found that Uncaria rhynchophylla (Miq.) Miq. ex Havil. uncarinic acid C extract (URC) enhanced the expression of CD40, CD38, CD1a, CD83, CD80, CD54, CCR7, HLA-DR and CD86 on DCs, and also promoted the production of IL-12p70 through TLR2 and TLR4 signaling pathways, leading to the conversion to Th1 subtype and the production of substantial amounts of IFN-γ. URC also prompted DCs to induce NF-κB transcription factor. DCs treatment with URC demonstrated moderate migration capability to CCL19 and CCL21 (95).

### 4.2 NF-κB pathway

The NF-κB pathway plays a pivotal role in the anti-tumor process, being a key downstream molecule of TLR4 (96). This pathway is composed of two branches: canonical and non-canonical pathways. And it is well known for its activity in responding to a series of external stimuli, such as immune response, cell proliferation, survival, and differentiation (97–100). Dysregulated NF-κB activity has implicated in inflammatory diseases and tumors, making it as a potential therapeutic target (101). IκB-α is the main regulatory protein that inhibits nuclear translocation. Numerous studies highlighted the interplay between NF-κB and MAPK signaling pathways in DCs activation and maturation (102). Studies have revealed that interferon (IFN) and NF-κB pathways were highly enriched in mature cDC1s associated with tumors. And the IFN response genes, which is necessary for anti-tumor immunity contained in cDC1, are regulated by IFN regulatory factor 1 (IRF1)-dependent NF-κB. Activated NF-κB promotes the recruitment and activation of CD8^+^T cells, which are important in orchestrating anti-tumor immunity (103).

E.-Q. L et al. found that Lycium barbarum Polysaccharides (LBP) induced the maturation of DCs, increased the production of IL-12p70 and IFN-γ, and promoted the expression of NF-κB in MLR, suggesting that these effects were associated with the NF-κB pathway (104). B. Zhao et al. discovered that a CHM formula Yangyinwenyang (YYWY) significantly inhibited tumor development in a mouse model of Lewis NSCLC. YYWY was found to promote DCs maturation through MAPK and MyD88-NF-κB pathways. This led to the generation of cytokines such as IL-2, IL-1β, IFN-γ, IL-12, and TNF-α from DCs. In addition, DCs matured under YYWY enhanced T cell proliferation and promoted differentiation of Th1 and CTL, thereby increasing the Th1/Th2 ratio (105).

### 4.3 MAPK pathway

MAPK pathway activation was a common feature in many types of cancers (106, 107). JNK, ERK and p38 MAPK pathways are often deregulated in cancers, and these pathways regulate the expression and activity of key inflammatory mediators, including proteases and cytokines, which may function as effective cancer promoters (108). Wang et al. found that inhibition of p38 MAPK restored the function, phenotype and cytokine secretion of BMDCs affected by tumor culture conditioning medium, thereby activating allospecific T cells, increased the expression of DC surface molecules, and enhanced the production of IL-12 (109).

T. Qin et al. modified polysaccharides extracted from Hericium ericium (HEP) to produce its nine selenium derivatives, sHEP1-sHEP9. Among them, sHEP1, sHEP2 and sHEP8 were found to increase the expression of MHC-II and CD86 on DCs, triggering their maturation. Additionally, sHEP2 and sHEP8 significantly reduced DCs endocytosis and strengthened the productions of cytokines (IL-12 and IFN-γ). Mechanistically, sHEP2 was found to promote the phosphorylation of JNK, p38 and ERK, as well as the nuclear translocation of p-c-Jun, p-CREB and c-Fos. Furthermore, sHEP2 also activated NF-κB pathway, leading to the decrease of the IκBα/β and nuclear translocation of p65 and p50 (110). Moreover, R. Yu et al. discovered that HEP also promoted DC maturation, with increased MHC II and CD86 expression, typical morphology, low antigen uptake, elevated TNF-α and IL-12 levels, and upregulation of MyD88, TLR4 and NF-κB proteins (111). D. H. Kim et al. found that 6-Acetonyl-5,6-dihydrosanguinarine (ADS) obtained from Chelidonium majus L. activated ERK/JNK phosphorylation in DCs by inducing ROS. This phosphorylation activated the NK-κB pathway, subsequently triggering the production of inflammatory cytokines, including TNF-α, IL-6, and IL-8 (112). J. Pan et al. found that Huaier extract enhanced the infiltration of CD4^+^ T cells and promoted DCs maturation in mice with 4T1 breast cancer. Huaier promoted the expression of co-stimulatory molecules in DC2.4 and BMDCs, as well as enhanced the levels of IL-12p70 and IL-1β, while inhibited their phagocytosis activities. Furthermore, Huaier promoted the proliferation of CD4^+^T cells, induced their differentiation into Th1 cells, which was achieved by modulating of the PI3K/Akt and MAPK pathways. Huaier increased the expression of p-Akt, Akt, PI3K, p-JNK, and JNK in BMDCs, while reduced the expression of p-p38 MAPK (113). N. Perera et al. found that the galactomannan isolated from Antrodia cinnamomea (ACP) stimulated the production of IL-6 and TNF-α in human monocyte-derived DCs through engagement with TLR4. ACP achieved this by activating MAPK and protein kinase C-α (PKC-α) phosphorylation, leading to the increased the phosphorylation of p38, JNK1/2 and ERK1/2. Furthermore, ACP induced TNF-α secretion and COX-2 expression via PKC-α (114).

### 4.4 JAK-STAT pathway

In addition, research has shown that CHM regulates DCs function through various signaling pathways, including the STATs pathway. The JAK/STAT pathway is particularly important in malignant tumors, with its inhibition holding therapeutic potential. One key regulator involved in the negative regulation of the JAK/STAT pathway is SOCS1, which exerts its effects through modulating phosphorylation states (115). JAK signals activate STAT-related transcriptional activity, driving gene expressions that control maturation, differentiation, and function in DCs (116).

In Chinese Herbal Medicine, there is a formula (SL) which consists of Lonicerae Japonicae Flos and Sophorae Flos. Y. X. Liu et al. found that treatments with an ethanolic extract of SL (SLE) increased the infiltrations of cytotoxic T cells (Tc), helper T cells (Th), and DCs in both melanoma tumors and spleens of mice. Furthermore, SLE was found to inhibit STAT3 activation in B16F10 cells, and to down-regulate the mRNA levels of STAT3 target genes in splenic lymphocytes (117). Y. Wang et al. discovered that the extract from Pinellia pedatisecta Schott plant, known as PE, had several beneficial effects on the immune response against tumors. They found that PE promoted the expression of MHC II and co-stimulatory molecules CD86 and CD80 on tumor-associated dendritic cell (TADCs), induced the production of IL-12 by TADCs, and enhanced the proliferation of both CD8^+^ T and CD4^+^ T cells. Moreover, PE induced the differentiation of GZMB^+^ CD8^+^ T and IFN-γ^+^ CD4^+^ T cells. *In vivo* experiments, PE strengthened the proliferation of cytotoxic T cells, as evidenced by increased expression of GZMB, CD137, Ki67, or TNF-α, IFN-γ. PE reversed the expression of PD-1 or CD95. The researchers confirmed that PE down-regulated SOCS1 expression and the phosphorylation of JAK2, STAT1, STAT4 and STAT5 in a time- and dose-dependent manner (118, 119).

### 4.5 Other pathways

J. Tian et al. found that Ficus carica Polysaccharides (FCPS) was able to effectively promote maturation of DCs through dectin-1/Syk pathway, leading to increased CD40, CD80, CD86 and MHC II expression. FCPS also stimulated DCs to produce cytokines, such as IL-6, IFN-γ, IL-12, and IL-23. Furthermore, FCPS enhanced the capacity of DCs to stimulate T cells and promoted T cell proliferation (120). F. Yao et al. discovered that Yupingfeng Granule (YPF) enhanced the proportion of mature DCs in tumors and adjacent tissues in mouse models of hepatocellular carcinoma. YPF treatment also led to a reduction in Th2 levels, an increase in Th1 levels, and an elevation in the Th1/Th2 ratio. *In vitro* experiments clarified that YPF not only promoted DCs maturation and stimulated IL-12 secretion, but also reduced the generation of OX40L and the ratio of CD4^+^ IL-13^+^ T cells. Of note, these effects were associated with the DC-mediated TSLP-OX40L pathway (121).

**Figure 1:**
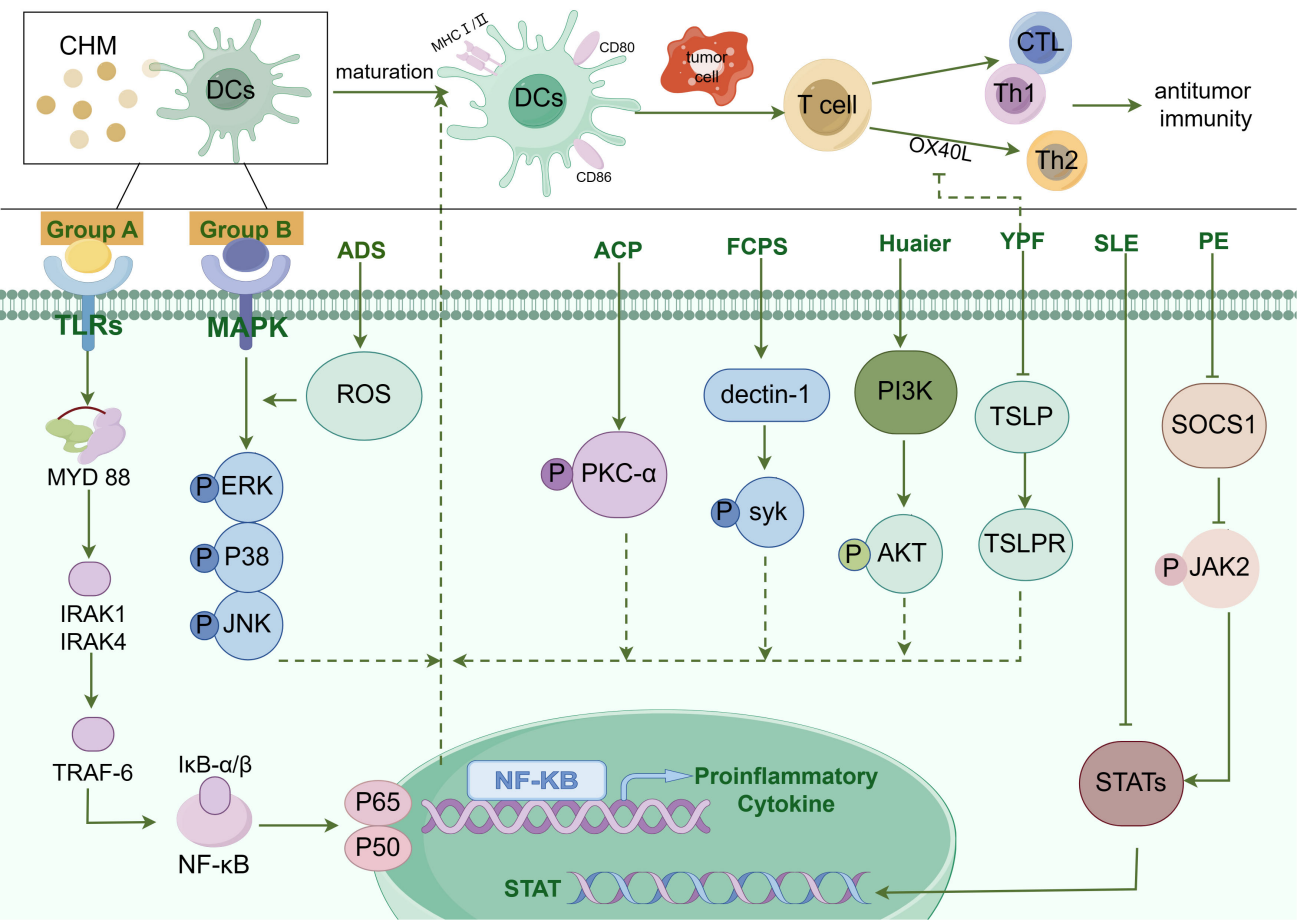
The diagram illustrating the pathways by which Chinese Herbal Medicines (CHM) promote DC maturation. The modulation of Dendritic Cell (DC) maturation by Chinese Herbal Medicine (CHM) encompasses a complex interplay of multiple signaling pathways, such as Toll-like receptor (TLR) pathway, NF-κB pathway, MAPK pathway, JAK-STAT pathway, and others. CHM compounds from group A, including Chrysanthemum coronarium L. (C. coronarium), the B-chain of Korean mistletoe lectin (KML-B), Actinidia eriantha Benth polysaccharide (AEPS), Lycium barbarum polysaccharides liposomes (LBPL), Matrine, Astragalus mongholicus (AMs), Astragalus polysaccharide (APS), Pleurotus ferulae water extract (PFWE), Uncaria rhynchophylla (Miq.) Miq. ex Havil. uncarinic acid C extract (URC), Yangyinwenyang (YYWY), Radix Glycyrrhizae polysaccharide (GP), and Hericium erinaceus polysaccharide (sHEP2), primarily regulate the NF-κB signaling pathway. Compounds in group B, such as Antrodia cinnamomea polysaccharide (ACP), Radix Glycyrrhizae polysaccharide (GP), Lycium barbarum polysaccharides (LBP), Hericium erinaceus polysaccharide (sHEP2), 6-Acetonyl-5,6-dihydrosanguinarine (ADS) and Huaier, predominantly modulate the MAPK signaling pathway. Additionally, Pinellia pedatisecta Schott extract (PE) mainly regulates the JAK/STAT pathway and contributes to DC maturation. Antrodia cinnamomea polysaccharide (ACP) promotes DC maturation by facilitating the phosphorylation of Protein Kinase C-α (PKC-α). Besides, DC maturation was also regulated by Syk, Akt, TSLP, and STAT3 pathways.

In conclusion, CHM promotes the functionality of DCs either directly or indirectly through various signaling pathways including MAPK, TLRs, NF-κB, or JAK-STAT. Here we summarize the pathway of CHM in regulating DC function in **Figure 1**. Mature DCs highly express MHC I and MHC II, CD80, CD86, and pro-inflammatory factors, activating the anti-tumor immunity. Currently, the majority of studies on signaling pathways which regulated DCs functionality are focused on NF-κB, MAPK, TLR, and JAK-STAT pathways. There is a need for further exploration of new signaling pathways induced by CHM in the maturation of DCs.

CHM does not exert its enhancing effect on the anti-tumor function of DCs via a singular signaling pathway. For instance, certain herbs, such as Radix Glycyrrhizae polysaccharide, may activate the MAPK pathway while also activating the NF-κB pathway. Currently, the majority of research focus on the regulatory effects of CHM on individual signaling pathways, while there is a lack of investigation into how CHM activates multiple signaling pathways simultaneously. In the future, utilizing gene chips or proteomics may enable researchers to elucidate the primary signaling pathways through which CHM enhances the anti-tumor functions of DCs.

CHM not only exert anti-tumor effects through the previously mentioned signaling pathways but are also capable of activating specific signaling pathways that suppress immune function. Utilizing the advanced techniques of gene chips or proteomics to investigate multiple signaling pathways, it is anticipated that researchers in the future may discover CHM monomers or compounds that selectively enhanced DC functionality through a specific activating signaling pathway.

## 5 Integration of traditional Chinese and western medicine

As science and technology advance, the integration of CHM with other treatments has emerged as a novel trend in anti-tumor research. For instance, the combination of CHM with Nanoparticles (NPs) or DCs-based Tumor Vaccines shows great promise.

NPs, as novel biomaterials, are increasingly being utilized across various medical fields. They promote anti-tumor effect by modulating DCs (122, 123). Targeted and sustained release of simple nanoliposomes containing Lycium barbarum polysaccharides (LBP) was employed as a potent immunological adjuvant, which effectively stimulate the proliferation of both CD4^+^ and CD8^+^ T cells *in vivo*, thereby enhancing the production of antibodies (124). The combination of lentinan with multiwalled carbon nanotubes exhibits low cytotoxicity, high solubility, and biological stability. It can rapidly enter DCs and significantly increase the expression of numerous antigens, thereby enhancing antigen-specific immunity (125). Zhang et al. found that Chinese yam polysaccharide-encapsulated poly (lactic-co-glycolic acid) (PLGA) nanoparticles had good stability, promoted antigen uptake of macrophages, increased MHC I, MHC II, CD86 and CD80 on DCs, whereby enhancing immune response (126). Studies have shown that Ganoderma lucidum polysaccharide combined with gold nanocomposites promoted the function of DCs in anti-tumor immunity, including the upregulation of MHC II, CD86, CD80, and pro-inflammatory factors transcription, as well as promoting the proliferation of CD8^+^ and CD4^+^T cells (127). The synergy between Alhagi honey polysaccharide and PLGA potentially enhanced its immune-modulating effects, strengthening the expression of MHC II and MHC I in DCs and boosting the proliferation of CD8^+^ and CD4^+^ T cells (128). Besides, Angelica sinensis polysaccharide bound to PLGA via the JAK2/STAT3 pathway, mediating DCs activation and maturation and enhancing the efficacy of the immune response (129). An investigation considered that Astragalus polysaccharide served as a potent adjuvant for cancer vaccination via DCs-mediated immunity (130). Song et al. demonstrated that Bai Hua She She Cao extract and its active ingredient rutin promoted antigen presentation and DCs activation through MAPK signaling pathway. The combination of Bai Hua She She Cao with peptide-based vaccines has been found to strengthen the immune response and improve the outcome for human papillomavirus-induced cancers (131).

Nanoparticles have shown promise in enhancing the efficacy of CHM, by safeguarding active components, diminishing cytotoxicity, and demonstrating robust targeting capabilities. However, several challenges persist. Firstly, the majority of existing research has focused on the integration of nanoparticles with individual CHM components, while clinical applications often benefit from the combined action of CHM compounds. The complexity of these compounds and the underdeveloped understanding of their pharmacodynamic mechanisms present significant hurdles for optimizing the anti-tumor effects of nanoparticle-based delivery systems. Second, the fabrication of nanoparticles containing CHM active ingredients lacks uniformity and standardization, which could affect their consistency and performance. Third, enhancing the targeting efficiency of nanoparticles while minimizing the risk of off-target effects is an area requiring further investigation. Lastly, although there have been numerous *in vitro* studies exploring the anti-tumor applications of nanoparticles in conjunction with CHM, these findings need to be corroborated through clinical trials to validate their efficacy and safety.

## 6 Clinical trial

In recent years, there has been a significant increase in clinical trials examining the application of CHM in anti-tumor therapies. Meta-analysis studies have shown that integration of CHM, such as Astragalus, with chemotherapy enhanced the response rate to treatment and reduced the incidence of adverse effects in patients with colorectal tumors (132). Ye et al.’s investigation has shown that the CHM preparation Shuangbai San effectively reduced pain in patients with primary liver cancer and improved their quality of life (133). Tian et al. found through an investigation of 60 non-small cell lung cancer patients that Feiji Recipe alleviated a series of adverse reactions caused by chemotherapy and improved their living standards (134). In a study involving 39 patients with advanced hepatocellular carcinoma, Changou and colleagues showed that the combination of PHY906 (made from Ziziphus jujube Mill, Paeonia lactiflora Pall, Glycyrrhiza urensis Fisch, and Scutellaria baicaliasis Georgi) and capecitabine enhanced anti-tumor efficacy (135). Ni and his team reported that Shenbai Granules, in a survey of 400 randomly assigned patients, were effective in reducing the recurrence rate of colorectal adenomas (136). Zhao et al. found through their study of 63 patients with esophageal cancer that Fuzheng Yiliu granules combined with radiotherapy enhanced immune response, and inhibited the metastasis and invasion of malignant tumors (137). Liu and colleagues conducted a comprehensive investigation involving 3483 patients with liver cancer and found that CHM remedy significantly prolonged patients’ survival and improved overall quality of life. Besides, it was also discovered that the universally used traditional Chinese patent medicines were Huaier Granule, Fufang Banmao Capsule, and Jinlong Capsule (138). Zhuang et al. found that in 105 cancer patients, CCMH (a mixture of extracts of A. sinensis, C. pilosula and G. lucidum and citronellol) combined with radiotherapy or chemotherapy maintained the numbers of neutrophils and leukocytes in blood, thereby improving immune function and enhancing anti-tumor efficacy (139). Liu et al. found through a study of 262 patients that compound Kushen injection significantly reduced symptoms such as cough, fatigue, and pain in lung cancer patients, alleviated radiation side effects, and improved their quality of life (140). Xiang et al. conducted a study involving 240 patients with advanced liver fibrosis and found that entecavir combined with traditional Chinese herbal formula (Ruangan granules) effectively alleviated liver fibrosis and prevented the occurrence of hepatocellular carcinoma (141). Xu et al. discovered that the combined treatment of modified Bu-zhong-yi-qi decoction and 5 fluorouracil suppressed the increase in CD8^+^PD-1^+^T cells caused by chemotherapy in gastric cancer patients, thereby inhibiting tumor immune escape (142). Jie et al. discovered that Sijunzi decoction upregulated the expression of Krüppel-like factor 4 in patients with colorectal cancers, thereby improving patients’ survival and reducing the recurrence rate of rectal cancers (143). Wu et al. discovered that Hezhong granules effectively alleviated nausea and vomiting caused by fluorouracil chemotherapy in advanced colorectal cancers, thereby improving their quality of life (144). Yang et al. found through a study of 291 liver cancer patients that the Fuzheng Jiedu Xiaoji formula restrained the migration and proliferation of liver cancer cells via the AKT/CyclinD1/p21/p27 pathway, improved patient survival, and reduced mortality (145). Small et al. found that PC-SPES (composed of eight herbal extracts) promoted the decrease of serum prostate-specific androgen and alleviated the progression of prostate cancer (146). Shao et al. found that Xiao Chai Hu Tang inhibited tumor growth by adjusting the TLR4/MyD88/NF-κB pathway mediated by gut microbiota (147). Zhao et al. conducted a study involving 361 cancer patients and found CHM prevented the recurrence of small hepatocellular carcinoma (148). Lou et al. found that Rhodiola algida combined with chemotherapy enhanced lymphocyte proliferation in breast cancer patients, strengthened their immunity and reduced the side effects following chemotherapy (149). Meng et al. discovered that GuizhiFuling Wan reduced the tumor burden in patients with uterine fibroids and alleviated related symptoms such as pain (150).

In clinical settings, the investigation into the anti-tumor effects of CHM has primarily centered on its therapeutic benefits, with less emphasis on the precise mechanisms driving its actions. Especially, there is a striking absence of research into how CHM influences DCs in the fight against tumors. On clinical trial databases, there are 114 entries involving CHM in anti-tumor treatments, yet only a single trial is currently active and enrolling patients, specifically targeting the interaction between CHM and DCs. This study is accepting patients with ovarian, colorectal, endometrial, and breast cancers, and it measures the production of IFN-γ, TNF-α, and IL-2 following CHM treatment. The researchers have established multiple platforms to assess the effects of CHM on modulating IL-17, PD-1/PD-L1, as well as to observe the activation of DCs and T cells. Hence, it is essential to allocate more time and resources to these experiments, in order to broaden the scientific foundation in this field.

## 7 Conclusion and perspectives

This accumulation of evidence suggests that various Chinese Herbal Medicines (CHM) and their active ingredients possess the capacity to development and function of antigen-presenting Dendritic Cells (DCs). These effects contribute to the anti-tumor properties of CHM. A summary of the influence of CHM-derived active ingredients and herbal compounds on DCs features is provided in **Table 2**. These studies demonstrate that CHMs can modulate the immune response by targeting DCs, emphasizing their therapeutic potential in treating immune disorders that are DC-dependent. This also suggests the feasibility of uncovering new biological modifiers of DCs from natural sources. However, further research is necessary to elucidate the molecular mechanisms underpinning the modulatory actions of CHM on DCs.

**Table 2:** The regulating effect of CHM on DCs.

Name	Molecular mechanism	Pathway	Ref.
Polysaccharides			
Alhagi honey polysaccharide (AH)	vitro experiments, ↑: pIgR protein in Caco-2 vivo experiments, ↑: CCL20, mDC, CD4^+^ T, CD8^+^ T, B, pIgR, J-chain, IgA^+^, sIgA, SCFAs		(58)
Sulfated Echinacea purpurea polysaccharide (EPP)	↑: CD11c, CD80, IFN-γ, IL-2 ↓: IL-4, IL-10		(59)
Lycium barbarum polysaccharides (LBP)	↑: MHC II and costimulatory molecules (CD80, CD86), IL-12p70, IL-4, IL-6 and IFN-γ	NF-kB; TLR4-Erk1/2-Blimp1	(85, 104)
Glycyrrhiza polysaccharide extract 1 (GPS-1)	vivo experiments, ↑: IFN-γ, IL-4, IL-10, CD3^+^CD4^+^ and CD3^+^CD8^+^ T vitro experiments, ↑: NO, IL-2, IL-1β, IFN-β, TNF-α and IL-12p70, IL-12, IFN-γ		(61) (60)
Radix Glycyrrhizae polysaccharide (GP)	↑: CD80, CD86 and IL-12 p70, MHC I-A/I-E, CD3^+^T, IFN-γ ↓: The endocytosis of FITC-dextran by DCs	p38 MAPK or JNK, TLR4, NF-κB	(86)
Acidic Epimedium polysaccharide (EPS-1)	↑: cytokine (TNF-α, IL-4, IFN-γ, IL-12 and IL-2); the vital surface molecules (CD86, CD40, CD11c and MHC II) and cytokine generation (TNF-α and IL-10) of matured chBM-DCs, phagocytic proportion of matured chBM-DCs		(62, 63)
Actinidia eriantha Benth polysaccharide (AEPS)	↑: cytokines (TNF-α, IL-1β, IL-6, IFN-β, IL-12p40, IL-10 and IFN-γ); chemokines (CCL5, MIP-1β, MIP-1α, MDC and MCP-1); pattern recognition receptors (DHX58, DDX58, TLR3 and IFIH1); ↓: phagocytic activity of BMDC	TLR2/4 and NF-κB	(87)
Hericium erinaceus polysaccharides (sHEP_1_-sHEP_9_)	sHEP_1_, sHEP_2_ and sHEP_8_: ↑MHC II and CD86, IL-12, IFN-γ sHEP_2_ and sHEP_8_: ↓DCs endocytosis	TLR4/MyD88/NF-κB	(110, 111)
Achyranthes bidentata polysaccharide (ABP)	↑: CD86, CD40, and MHC II, IL-12		(64)
Isatis root polysaccharide (IRPS)	↑: the maturation of MoDCs, IL-12, IL-10, IL-1β, and IL-12p35, IFN-γ, TNF-α ↓: IL-6		(65)
Rehmannia glutinosa polysaccharide (RGP)	↑: IL-2, IFN-γ, T lymphocytes, the antigen presenting ability		(66)
Ficus carica polysaccharides (FCPS)	↑: CD86, CD80, CD40, IFN-γ, IL-12, MHC II, IL-6, and IL-23, T cells	dectin-1/Syk	(120)
Antrodia cinnamomea polysaccharide (ACP)	↑: TNF-α, IL-6	PKC-α MAPK	(114)
Astragalus mongholicus (AMs)	↑: TLR4 ↓: IκB-α	TLR4	(90)
Astragalus polysaccharide (APS)	↑: CD86, CD80, CD40, MHC I/II, IL-6, IL-12p70 and TNF-α	TLR4-MyD88	(32, 91, 92)
Plantain polysaccharide (PLP)	↑: mDC, naive T cells into cytotoxic T cells		(67)
Saponins			
Ginsenoside-Rg1 (G-Rg1)	↓: IDO1 ↑: mDC		(70)
Astragaloside IV (AS-IV)	↑: mDC, IL-12, CD80, CD40, CD14, HLA-DR, CD86, and CD83		(71)
The water extract			
Chrysanthemum coronarium L. (C. coronarium) water extract	↑: CD40, CD86, MHC I, MHC II, IL-12, IFN-γ	TLR2, TLR4 and TLR9	(84)
Pleurotus ferulae water extract (PFWE)	↑: CD86, CD80, CD40 and MHC II; IL-6, IL-12 and TNF-α	TLR4	(93)
The acid extract			
Uncaria rhynchophylla ursolic acid extract	↑: CD1a, CD86, CD83, CD80, HLA-DR and CCR7, IL-12: enhanced the T cell stimulatory capacity in an allogeneic MLR	TLR2 and TLR4	(94)
Uncaria rhynchophylla (Miq.) Miq. ex Havil. uncarinic acid C extract (URC)	↑: CD40, CD38, CD1a, CD83, CD80, CD54, CD86, HLA-DR and CCR7, IL-12, IFN-γ; stimulated naïve T cells into typical Th1 cells	TLR2 and TLR4	(95)
Chinese herb medicine compound			
Yangyinwenyang (YYWY)	↑:IL-2, IL-1β, IFN-γ, IL-12, CTL, T cells (Th1), TNF-α, Th1/Th2 (IFN-γ/IL-4 radio)	MAPK and NF-kB	(105)
Shi-Quan-Da-Bu-Tang (SQDBT)	↑: antigen presentation to MHC I in DC2.4 cells and original DC cells		(76)
Hochu-ekki-to (Bu-Zhong-Yi-Qi-Tang) (HOT)	↑: MHC II, co-stimulatory molecules (CD40, CD80 and/or CD86) and IL-12		(72, 77)
Yupingfeng Granule (YPF)	vitro experiments, ↑: mDC, Th1, Th1/Th2, CD80, CD86, MHC II vivo experiments, ↓: TSLP, TSLPR, OX40L, Zis, Th2, IL-4, IL-10, IL-5 vitro experiments, ↑: mDC, IL-12 vivo experiments, ↓: OX40L, CD4^+^ IL-13^+^ T, Th2	TSLP-OX40L	(121)
Huangqi Guizhi Wuwu Tang (HGWT)	↓: IL-6, IL-12 p40		(68)
Others			
Salidroside liposome	↑: promote DC maturation, proliferation, antigen presentation capacity CD80, CD86, MHC II, MHC I; prolong the content of IL-2, IFN-γ, IgG in the serum.		(69)
Lycium barbarum polysaccharides liposomes (LBPL)	↑: co-stimulatory molecules (CD86, CD80, MHC II), production of cytokines (IL-12p40, TNF-α);	TLR4-MyD88-NF-κB signaling pathway	(88)
Cordyceps Sinensis	↑: costimulatory molecules (CD40, CD80, CD86), T cells		(75)
B-chain of Korean mistletoe lectin (KML-B)	↑: CD86, CD80, CD40, MHC II, IL-12p70, IL-6, IL-1β, TNF-α	TLR4	(83)
Myrothecine A	↑: CD86, CD40		(73)
Plantago asiatica L. seeds extract (ES-PL)	↑: CD86, CD80, MHC II, *CCR7* ↓: mannose receptor-mediated endocytosis		(74)
Huaier	↑: mDC, CD4^+^T, Th1 *In vitro* experiments: ↑costimulatory molecules of DC2.4 and BMDCs; IL-1β IL-12p70, ↓: phagocytic activities	MAPK and PI3K/Akt	(113)
Matrine	↑: TNF-α, IL-6, IL-12, MHC-II, CD54, CD80, CD86	TLRs	(89)
6-acetonyl-5,6-dihydrosanguinarine (ADS) of Chelidonium majus L.	↑: TNF-α, IL-6, and IL-8	ROS-ERK/JNK-NF-kB	(112)
Pinellia pedatisecta Schott extract (PE)	↑: MHC II, CD80 and CD86, IL-12, CD4^+^ and CD8^+^ T cells, GZMB, CD137, Ki67 or IFN-γ, TNF-α; the differentiation of GZMB^+^CD8^+^ T and IFN-γ^+^CD4^+^ cells; CD107a, GZMB, and perforin in CTLs ↓: SOCS1	SOCS1 JAK2-STAT1/STAT4/STAT5	(118, 119)
SL ethanolic extract (SLE)(SL:A Chinese Herbal Medicine formula comprising Sophorae Flos and Lonicerae Japonicae Flos)	↑: Th, Tc, DC	STAT3	(117)

"↑" indicates an increase in the previously mentioned factors, and "↓" indicates a decrease in the previously mentioned factors.

Despite advancements, certain challenges persist in the study of CHM and its interactions with DCs in combating tumors. Firstly, existing research primarily concentrates on the quantity and activity of DCs, while investigations into the impact of CHM on the subset classification of DCs and the associated signaling pathways are notably lacking. Secondly, the lack of standardized and systematic processing of CHM, along with the separation and purification of active ingredients, significantly affects DC functionality and the anti-tumor effect as certain unwanted compounds within CHM have been identified to possess immunosuppressive effects, mitigate DCs maturation and the production of pro-inflammatory cytokines. This observation highlights the need for future research to concentrate on understanding the implications of these compounds and their impact on the immune response. Clinical research on CHM has predominantly been conducted on the Chinese population, which lacks global representation, randomization, and a systematic approach. Furthermore, most clinical studies focus on anti-tumor efficacy, often overshadowing the in-depth exploration of the underlying anti-tumor mechanisms involving DCs. To validate the influence of CHM on DCs, conditional knockout mice lacking DCs could be utilized. With the growing recognition of DCs’ pivotal role in the immune response, there has been a surge in research on DC-based vaccines for tumor treatment, while the integration of CHM with DC vaccines for tumor therapy presents an exciting avenue for future research.

CHM augments anti-tumor efficacy through the modulation of DCs, aligning with the CHM principles of “strengthening the body’s defenses to expel pathogens” and “maintaining balance between Yin and Yang.” This action can be likened to the revitalizing the body’s immune system, refining the extracellular matrix’s internal environment, and subsequently eliciting anti-tumor effects. However, the specific role of DCs in mediating the anti-tumor effects of CHM and its underlying mechanisms remain elusive. In this review, we provide a comprehensive summary of the molecules and the precise mechanisms through which CHM interfaces with DCs. Our objective is to provide researchers with a comprehensive roadmap for future exploration. CHM holds the potential to facilitate the advancement of enhanced protocols for DCs maturation, thereby paving the way for improved cancer treatment outcomes and enhanced quality of life for patients.
